# Phalangeal bone growth and implications in Turner syndrome

**DOI:** 10.3389/fendo.2025.1735962

**Published:** 2026-01-12

**Authors:** Min Jae Kang, Roopa Kanakatti Shankar, Youn Hee Jee

**Affiliations:** 1 Department of Pediatric Endocrinology, Children’s National Hospital, Washington, DC, United States; 2 Department of Pediatrics, Hallym University Sacred Heart Hospital, Hallym University College of Medicine, Chuncheon-si, Gangwon-do, Republic of Korea; 3 The George Washington University School of Medicine and Health Sciences, Washington, DC, United States

**Keywords:** bone age X-ray, brachydactyly type A3, metacarpal bone, middle phalanx, SHOX deficiency, Turner syndrome

## Abstract

**Purpose:**

Skeletal abnormalities are common in Turner Syndrome (TS), yet data on objective radiographic markers are limited. We aimed to establish normative reference ranges for phalangeal length ratios and assess their utility in detecting skeletal abnormalities in TS.

**Methods:**

We analyzed 4,082 female bone age X-rays (<18 years) from the Radiological Society of North America (RSNA) database after quality screening and outlier exclusion as a reference cohort. Phalangeal length ratios—4th to 3rd metacarpal (4:3 MC), 5th to 3rd metacarpal (5:3 MC), and 5th to 3rd middle phalanx (5:3 MP)—were measured and compared in 81 TS patients seen at a single center. Additional skeletal features such as *SHOX* deficiency-related signs and brachydactyly type A3 (BDA3) were assessed.

**Results:**

In reference subjects, 4:3 MC and 5:3 MC ratios remained stable across most age groups, while the 5:3 MP ratio increased with age. TS patients showed a significantly lower 4:3 MC and 5:3 MP ratios (P < 0.001, P = 0.002, respectively) compared to ones from reference subjects. A low 4:3 MC ratio (<–2 SD) was seen in 27.2% of TS patients. The 4:3 MC ratio correlated with height percentile (r = 0.27, P = 0.02). BDA3 was more prevalent in TS compared to reference subjects (13.6% vs. 2.1%, P < 0.001) and associated with low MC ratios.

**Conclusion:**

Normative reference ranges for phalangeal length ratios were established and differences in 4:3 and 5:3 MP ratios in patients with TS were identified compared to the reference group. Further studies with larger TS cohorts are needed to confirm the clinical utility of these radiographic biomarkers.

## Introduction

Bone age assessment, typically performed using an X-ray of the left hand, is a widely used tool to evaluate skeletal maturation in children (1). First developed by Drs. Greulich and Pyle in the early 20th century (2), bone age remains a standard method for assessing growth, pubertal progression, and pathological growth conditions (3). Beyond its use in estimating skeletal age, bone age X-rays have been instrumental in studying tubular bone growth patterns in children. For instance, standing height has been shown to correlate strongly with the lengths of the phalanges (4). In many genetic syndromes, the lengths and proportions of tubular bones on hand X-rays are notably altered, making these features potentially valuable for diagnosis (5, 6). As early as the 1980s, studies tested metacarpophalangeal patterns to identify characteristic features associated with various malformation syndromes (5, 6). Both absolute bone lengths and relative ratios—such as the index-to-ring finger length ratio—have been used to infer clinical and biological information. For example, this ratio has been proposed as a marker of fetal sex (7) or a marker of the osteoarthritis risks (8).

Turner syndrome (TS), a well-known chromosomal disorder affecting skeletal development, provides a compelling example of how bone age X-rays can reveal characteristic skeletal abnormalities (9–11). Most individuals with TS have a deficiency in the Short Stature Homeobox (*SHOX*) gene, leading to a set of typical wrist findings such as medial epiphyseal lucency of radius, a malpositioned lunate between V-shaped radial and ulnar epiphyseal lines, and pyramidalization of the radial epiphysis (12). In addition to short metacarpals, recent studies have identified abnormal tubular bone ratios in TS, such as the distal-to-middle phalanx ratio of the fifth digit (13) and the height-to-width ratio of the fourth metacarpal (14). One study reported that the ratio of the fifth digit’s distal to middle phalanx could distinguish 98 patients with TS from 96 controls (13).

However, it remains unclear how frequently these radiographic features occur among individuals with TS, or whether specific phalangeal or metacarpal ratios can serve as reliable diagnostic tools. Moreover, previous studies have been limited by the lack of a large reference dataset of bone age X-rays from the general population providing an insight of growth in these bones. Therefore, the primary aim of this study was to establish normative reference ranges for phalangeal and metacarpal ratios—the ratios of the 4th to 3rd metacarpals, the 5th to 3rd metacarpals, and the 5th to 3rd middle phalanges—using a publicly available dataset of bone age X-rays in females from the Radiological Society of North America (RSNA) (15). These reference values were compared to measurements from patients with Turner syndrome to better understand differences in long bone growth. The secondary aim was to investigate potential associations between bone age abnormalities and clinical growth parameters in patients with TS.

## Subject and methods

### Bone age X-rays for reference data

**Figure 1:**
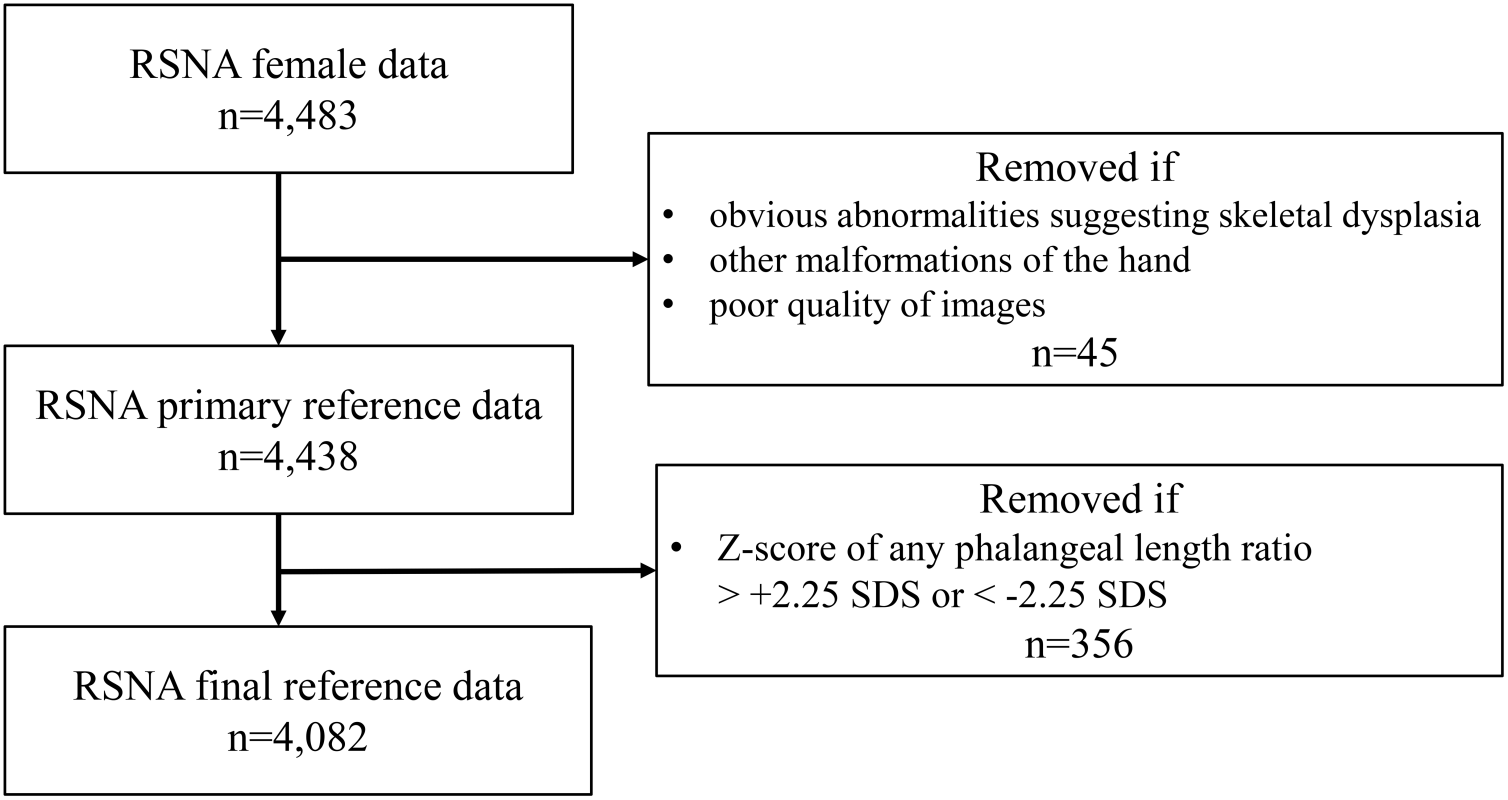
Diagram of bone age X-ray inclusion and exclusion from the Radiological Society of North America (RSNA).

We utilized 4,483 bone age X-rays (from females under 18 years of bone age) and corresponding bone age data obtained from the RSNA dataset (15). The RSNA dataset does not provide normative data, nor are there published normative values derived from it. Therefore, our research team reviewed the images and measured the phalangeal ratios to establish normative data. Each image was anonymized and coded upon download. Two experienced pediatric endocrinologists (with over 15–20 years of experience interpreting bone age X-rays) independently reviewed the images and excluded bone age X-rays with apparent abnormalities suggestive of skeletal dysplasia, malformations, or poor image quality. Of the 4,483 reviewed images, 45 (1%) were excluded, resulting in a preliminary reference set of 4,438 X-rays (**Figure 1**). We chose to study the 3rd, 4th, and 5th metacarpals (MC) and the 3rd and 5th middle phalanges (MP) because they have traditionally been examined when evaluating phalangeal growth and patients with TS also have a high prevalence of abnormal bone development in the 4th and 5th metacarpals (11). To maintain consistency, all phalangeal length measurements were performed by a single endocrinologist. Due to variations in image magnification, relative ratios of phalangeal lengths, rather than absolute measurements, were used for analysis. The phalangeal ratios in the RSNA dataset were normally distributed. To ensure a reliable reference range because clinical information was unavailable, outliers at the tail ends of the distribution (i.e., subjects with Z-score below -2.25 or above +2.25 standard deviations (SDS)) were further excluded to generate a robust normative dataset. The final reference dataset comprised 4,082 bone age X-rays.

### Turner syndrome dataset

**Figure 2:**
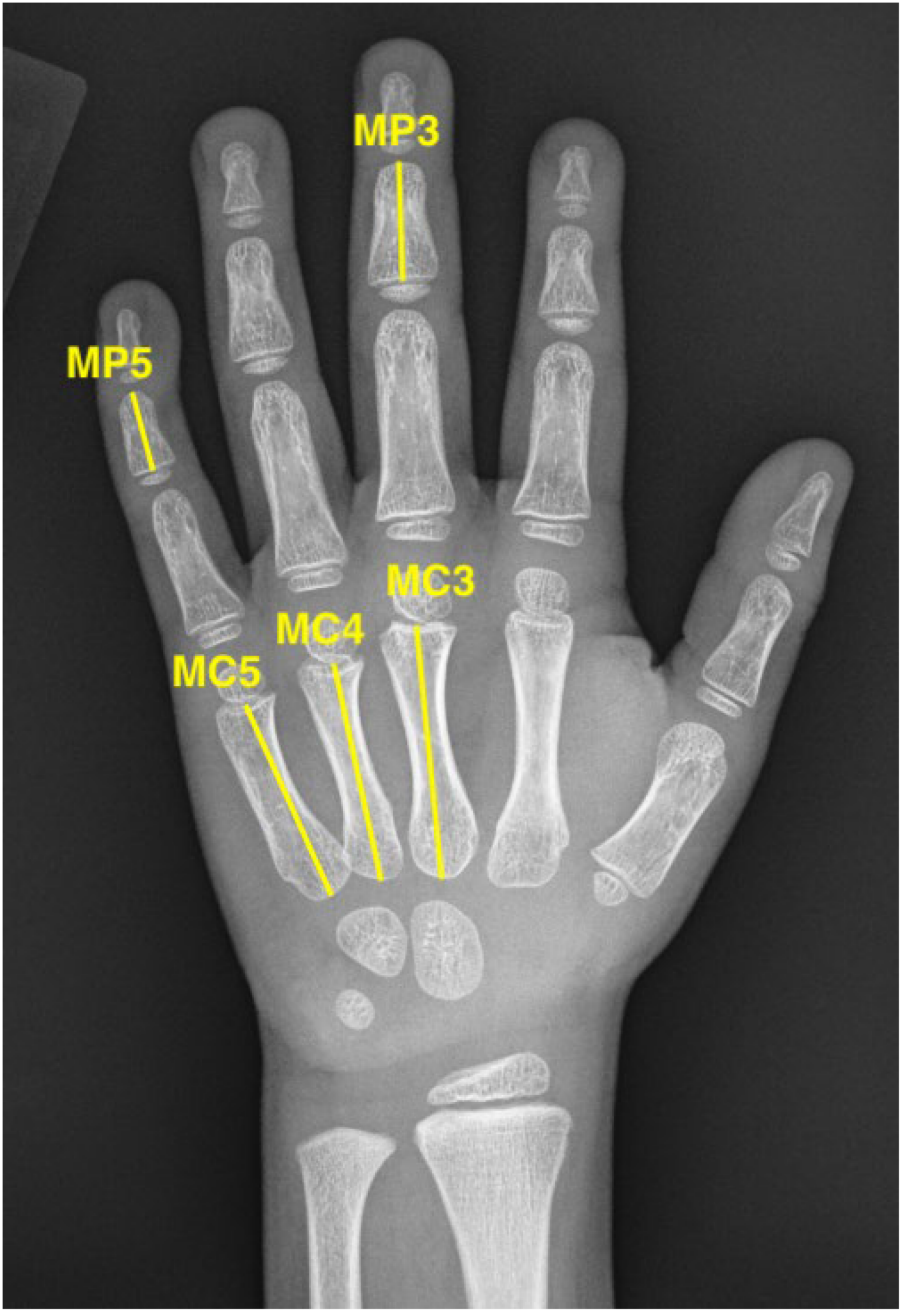
Measurement of 3^rd^, 4^th^, and 5^th^ metacarpals and 3^rd^ and 5^th^ middle phalanges. MP5, 5^th^ middle phalanx. MP3, 3^rd^ middle phalanx. MC5, 5^th^ metacarpal. MC4, 4^th^ metacarpal. MC3, 3^rd^ metacarpal.

Eighty-one bone age X-rays from female patients with Turner syndrome (TS), ages 3.0–17.9 years, were analyzed. These patients were seen in a multidisciplinary clinic at Children’s National Hospital. The study was reviewed by the Institutional Review Board (Protocol Pro00016829) and certified as exempt. A waiver of informed consent and assent was granted for the retrospective data analyses. For both the reference and TS groups, subjects were stratified into six age groups aligned with bone ages corresponding to pubertal developmental stages. Group 1: 0–5.9 years (young childhood), Group 2: 6.0–7.9 years (prepubertal), Group 3: 8.0–10.9 years (early pubertal), Group 4: 11.0–12.9 years (midpubertal), Group 5: 13.0–14.9 years (late pubertal), Group 6: 15.0–18.0 years (postpubertal) (**Supplementary Table S1**). For TS patients, clinical data were extracted from electronic health records, including karyotype, anthropometric measurements, final adult height (if reached), and growth hormone (GH) treatment status. Phalangeal lengths were measured for the following bones: 3rd, 4th, and 5th metacarpals (MC), and 3rd and 5th middle phalanges (MP). Measurements were taken from the midpoint of the base to the midpoint of the distal end of each bone (16), using ImageJ software (version 1.54i) (**Figure 2**). The following ratios were calculated: 4th MC to 3rd MC (4:3 MC), 5th MC to 3rd MC (5:3 MC), and 5th MP to 3rd MP (5:3 MP). Common skeletal abnormalities were independently assessed by two pediatric endocrinologists. Definitions were standardized prior to image review. For example, radiological signs of *SHOX* deficiency were defined as follows (12): i) Triangularization of the radial epiphysis only when capping of other epiphysis is observed (young bone ages show triangularization of the radial epiphysis normally until capping begins), ii) pyramidalization of carpal bones when lunate is growing into the space forming a wedge-shaped carpal row, and iii) Radiographic radial lucency when reduced bone density on the ulnar side of the distal radius is observed. Other defined abnormalities included: Brachydactyly Type A3 (BDA3) when 5:3 MP ratio < 0.5 or short metacarpals: 4:3 or 5:3 MC ratio < -2 SD compared to age-matched RSNA reference values.

### Statistical analysis

All data were analyzed using IBM SPSS Statistics (Version 29.0). Variables were log-transformed where necessary. ANOVA with Scheffé *post-hoc* tests was used to compare phalangeal ratios among age groups in the reference and TS dataset. Pearson’s correlation assessed relationships between bone age and phalangeal ratios. Linear regression controlled for age effects were tested for analyzing the association between phalangeal ratios and clinical parameters. Chi-square or Fisher’s exact test was used to compare categorical variables as appropriate. Student’s t-test compared continuous variables between two independent groups. Receiver operating characteristic (ROC) curve analysis was used to assess the diagnostic utility of the 4:3 MC ratio in distinguishing TS from the reference. The Youden Index determined the optimal cut-off value. A two-sided p-value < 0.05 was considered statistically significant. Corresponding graphs were generated using OneNineAI software, available at (https://onenine.ai/).

## Results

### Reference values for phalangeal length ratios

**Figure 3:**
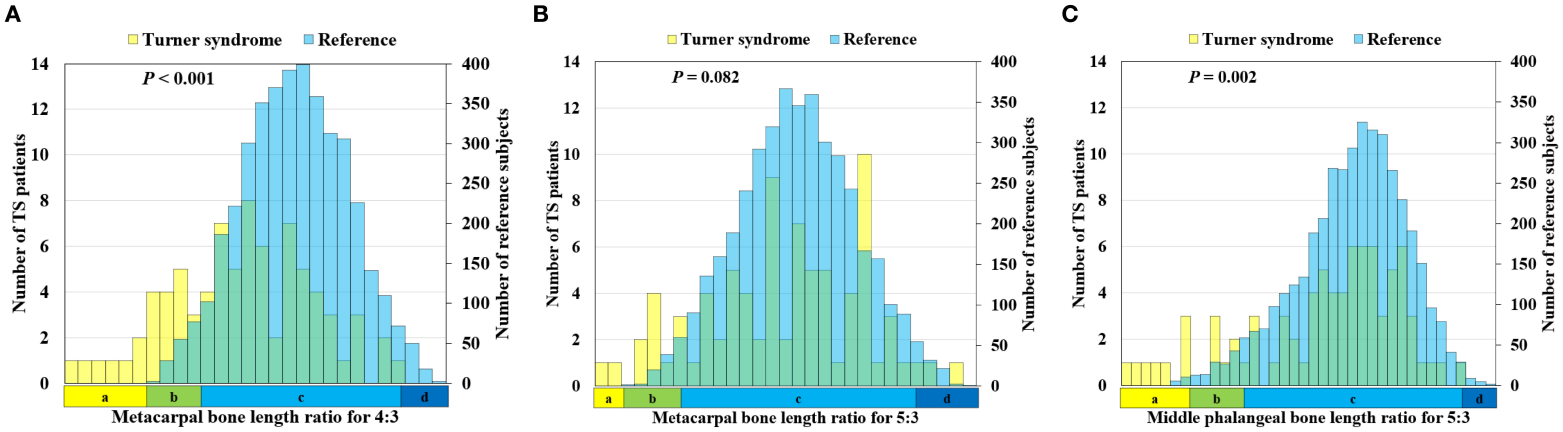
Distribution of ratios in the reference data and Turner syndrome (TS). **(a)** The 4:3 metacarpal ratios. **(b)** The 5:3 metacarpal ratios. **(c)** The 5:3 middle phalangeal ratios. In the X-axis, ‘a’ represents values from the minimum value to -3 SD of the reference, ‘b’ represents -3 SD to -2 SD of the reference, ‘c’ represents -2 SD to +2 SD of the reference, and ‘d’ represents +2 SD of the reference to the maximum value.

**Figure 4:**
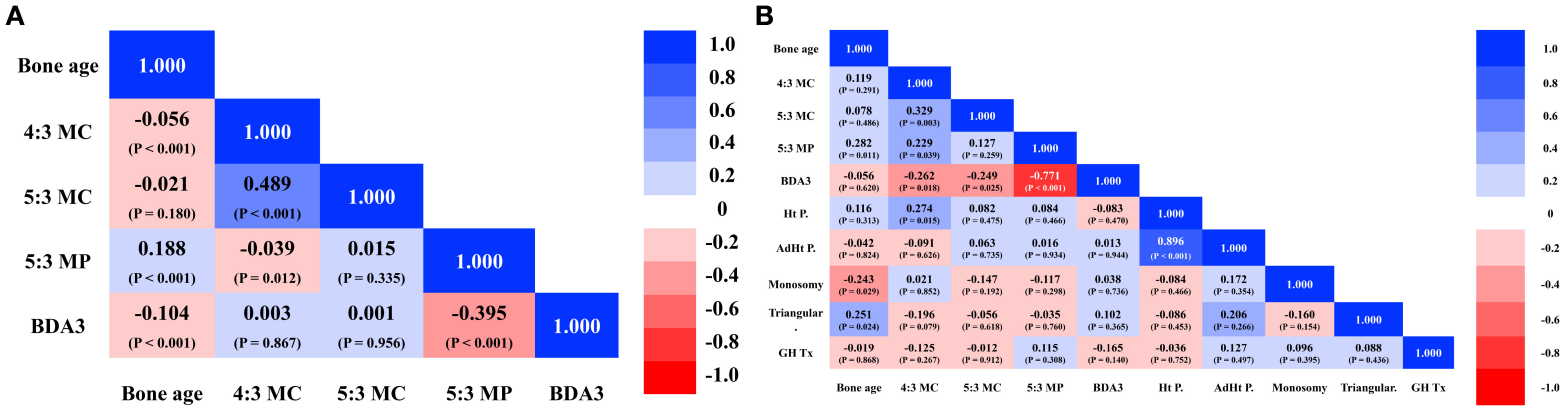
**(a)** Correlation heatmap, including 3rd, 4th, 5th metacarpals (MC), 3rd, 5th middle phalanges (MP), bone age and brachydactyly type A3 (BDA3) in the reference group. Blue indicates positive association, and red color indicates negative association. Each value indicates r with the corresponding P value in parenthesis. **(b)** Correlation heatmap, including 3^rd^, 4^th^, 5^th^ metacarpals (MC), 3rd, 5th middle phalanges (MP), bone age, brachydactyly type A3 (BDA3), height percentile (Ht P.), adult height percentile (AdHt P.), monosomy, triangular radial epiphysis, and growth hormone treatment (GH Tx) in TS. Blue indicates positive association, and red color indicates negative association. Each value indicates r with the corresponding P value in parenthesis. https://onenine.ai/.

Across age groups 1 to 5, the 4:3 MC and 5:3 MC ratios remained stable. However, both ratios were significantly lower in age group 6 (all pairwise comparisons, P < 0.001) (**Supplementary Figure 3A**), suggesting early growth plate closures of the 4th and 5th MCs than the 3rd MC during late puberty. Conversely, the 5:3 MP ratio was significantly higher in age groups 5 and 6 compared to groups 1–4 (P < 0.001), indicating a possible delay in 5th MP growth plate closure or earlier growth deceleration of the 3rd MP (**Supplementary Figure 3A**). All three phalangeal length ratios in the reference dataset followed a normal distribution, as indicated by skewness/kurtosis (4:3 MC: 0.002/–0.420; 5:3 MC: 0.018/–0.359; 5:3 MP: –0.503/0.088) (**Figures 3A–C**). The mean ± SD for the 4:3 MC and 5:3 MC ratios were 0.889 ± 0.019 and 0.822 ± 0.022, respectively (**Supplementary Table S2**). A moderate positive correlation was observed between the 4:3 MC and 5:3 MC ratios (r = 0.49, P < 0.001) (**Figure 4A**). There was no significant difference in the 5:3 MP ratio between groups 5 and 6 (P = 0.39). Although the 5:3 MP ratio showed a weak positive correlation with bone age (P < 0.001), the small r² value indicated a limited association. Additionally, the 4:3 MC did not correlate with the 5:3 MC ratios or the 5:3 MP ratio (**Figure 4A**).

### Phalangeal length ratios in Turner syndrome

Among the 81 TS patients, 33 had a 45,X karyotype and 48 had other karyotypic variants (e.g., ring chromosome, isochromosome). In patients with TS, the mean ± SD values for the 4:3 MC, 5:3 MC, and 5:3 MP ratios were 0.860 ± 0.050, 0.820 ± 0.040, and 0.600 ± 0.090, respectively. The 4:3 MC and 5:3 MP ratios were significantly lower than those in reference subjects (P < 0.001 and P = 0.002, respectively) but not the 5:3 MC ratios (**Figures 3A–C**). The prevalence of a low 4:3 MC ratio (defined as < –2 SD of age-matched RSNA reference) was 27.2%, higher than the prevalence of low 5:3 MC (13.6%) or 5:3 MP (16.0%) ratios in the TS group. Analysis of older bone age (≥ 8 years) did not increase the prevalence of abnormal ratios or other skeletal abnormalities (**Supplementary Table S3**). While the 4:3 MC and 5:3 MC ratios remained stable across age groups, the 5:3 MP ratio increased modestly with age (r = 0.28, P = 0.011), with a significant difference between age groups 2 and 6 (P = 0.02). In TS patients, different from the reference population, the 4:3 MC ratio was positively correlated with both the 5:3 MC (r = 0.478, P = 0.005) and 5:3 MP ratios (r = 0.26, P = 0.02) (**Figure 4B**). Receiver operating characteristic (ROC) analysis of the 4:3 MC ratio showed an AUC of 0.72 (95% CI: 0.656–0.783) and the optimal cut-off (Youden index = 0.88) had a sensitivity of 61.7% and specificity of 75.1%. The AUCs for the 5:3 MC ratio and 5:3 MP ratio were 0.54 (95% CI: 0.47-0.61) and 0.59 (95% CI: 0.52-0.66), respectively, indicating no significant diagnostic value.

### Clinical correlations in TS

Karyotype classification (45,X vs. other karyotypes) was not associated with phalangeal ratios, height percentile, adult height, or skeletal abnormalities. The 4:3 MC ratio showed a significant positive correlation with height percentile (r = 0.27, P = 0.02) after adjusting for bone age. No such correlation was found for the 5:3 MC or 5:3 MP ratios. In the TS cohort, 82% (n = 65) received GH therapy, which was not associated with differences in phalangeal ratios (**Figure 4B**).

### Other skeletal abnormalities in TS

#### *SHOX*-related features

The prevalence of triangularization, lucency, and pyramidalization in TS patients was 33.3%, 23.5%, and 13.6%, respectively. Triangularization was positively associated with bone age (r = 0.251, P = 0.024), and its prevalence increased as bone ages get older (**Supplementary Table S3**). None of the *SHOX*-associated features correlated with phalangeal ratios, height SDS, adult height SDS, or GH treatment status.

#### Brachydactyly type A3

The prevalence of BDA3 (5:3 MP ratio < 0.5) was significantly higher in TS patients (13.6%) than in the reference group (2.1%; 86 of 4,082 subjects) (P < 0.001). In reference subjects, the prevalence of short 4:3 or 5:3 MC ratio was not correlated to the prevalence of BDA3. However, in TS patients, BDA3 was associated with a higher prevalence of abnormal MC ratios: low 4:3 MC (54.2% vs. 22.9%, P = 0.061) and low 5:3 MC (36.4% vs. 10.0%, P = 0.038). Our findings indicate that the bone growth of phalanges differs in TS compared to the reference population. However, BDA3 did not correlate with height percentile, adult height, or other skeletal abnormalities in TS.

## Discussion

Our study is the first to present reference data on phalangeal length ratios in bone age group-specific female pediatric subjects, suggesting developmental trends in phalangeal bone growth. In the RSNA reference population and TS patients, we found that the 4:3 and 5:3 metacarpal (MC) ratios remained relatively stable across bone age groups (similar rate of bone growth and growth plate closure), while the 5:3 middle phalanx (MP) ratio increased with bone age (different rate of bone growth and growth plate closure). Although absolute phalangeal lengths are known to increase with growth, data on phalangeal length ratios are limited (5, 17) and inconsistent (18, 19). Overall, the 4th or 5th MCs reached growth plate senescence earlier than the 3rd MC, while the 5th MP does not seem to reach growth plate senescence earlier than the 3rd MP in the RSNA dataset, suggesting distinct regulatory mechanisms for metacarpal and phalangeal growth and senescence (**Supplementary Figure 3A**). Phalangeal ratios in TS were lower than in controls, especially the 4:3 MC ratio, with a 27.2% prevalence of low 4:3 MC ratio in TS patients, consistent with prior reports (20, 21). Although both 4th and/or 5th MC shortening (metacarpal sign) are observed in TS, the 5:3 MC ratio did not significantly differ from controls, and its correlation with the 4:3 MC ratio was weak (**Figures 3A–C**). Receiver operating characteristic (ROC) analysis of the 4:3 MC ratio showed an AUC of 0.72 (95% CI: 0.656–0.783) had a low sensitivity of 61.7% and specificity of 75.1%, indicating the 4:3 MC ratio is probably not a sensitive marker for TS. Given that the metacarpal sign is associated with gonadal dysgenesis (22) and short stature (23), we explored correlations between 4:3 MC ratio and height percentile. We observed only a weak positive correlation and a low r² value indicating a limited predictive utility. Our findings indicate that frequent abnormal phalangeal ratios in TS do not predict overall long bone growth deficit in TS. In addition, our data suggests that short MC and MP ratios in TS may be due to poor growth of disproportionate growth or different growth plate closure of each phalanx. Future study with a bigger TS patient cohort may address this more confidently.

Monosomy (45,X) is associated with more severe phenotypes in TS (24), but we found no differences in phalangeal ratios, skeletal features, or height percentile between monosomy and other karyotypes, consistent with prior studies (11). Brachydactyly type A3 (BDA3) was significantly more common in TS (13.6%) than in reference subjects (2.1%), consistent with previous reports (25, 26). In TS, low metacarpal ratios were associated with BDA3, suggesting that the underlying mechanisms—such as impaired bone growth or premature growth plate closure—may be shared among the 4th MC, 5th MC, and 5th MP. This is supported by our observation that a positive correlation between the 4:3 MC and 5:3 MP ratios is only found in TS but not in reference subjects. We suspect that not only shortening of the 4th and/or 5th metacarpals but also the 5^th^ MP may be attributed to premature growth plate closure driven by *SHOX* deficiency. Among other *SHOX*-related features, triangularization was most common and appeared more frequently with age, supporting its age-related progression (12).

The majority of TS in our cohort received GH treatment. We observed no clear associations between GH treatment and changes in phalangeal ratios. Although we observed a slightly higher prevalence of low 5:3 MP ratios in untreated patients, raising the possibility that GH may influence small bone growth, it requires a future longitudinal study for confirmation.

Our study has several limitations. First, bone lengths were measured manually, and variations in epiphyseal morphology or the angulation of tubular bones may have introduced measurement bias. In future studies, machine learning-assisted image analysis could provide more objective and precise assessments of bone lengths and ratios. Second, although previous studies have suggested that hand preference may influence phalangeal growth (17, 27), we had no data on hand dominance and analyzed only left-hand bone age X-rays. Third, while we proposed a cut-off value of 0.876 for the 4:3 MC ratio in diagnosing TS, its sensitivity and specificity were suboptimal—which might be due to a small number of TS patients in our cohort. This may change in larger studies. Fourth, the RSNA dataset does not include subjects’ height measurements or diagnostic information, nor provides it record the clinical indications for obtaining a bone age X-ray. As a result, the potential impact of these factors on the normative data could not be assessed in the current study. An expanded analysis using a large institutional dataset with comprehensive clinical information is underway to address these limitations. Fifth, the most ideal reference range should be established with data from healthy children of different ages, which is not feasible. To mitigate this limitation, we excluded X-rays with obvious abnormalities as well as extreme measurements (<–2.25 or >+2.25 SD) to ensure that the resulting reference range approximates a true normative sample as closely as possible. Lastly, most patients in our TS cohort were treated with GH, and therefore our dataset cannot adequately assess the impact of GH on phalangeal growth. Turner syndrome has been an approved indication for GH therapy since 1996, and most patients begin treatment shortly after diagnosis. As a result, it is unlikely that sufficiently robust data from untreated TS patients can be obtained, as very few remain untreated in current clinical practice. Consequently, the small size of the untreated group in our cohort limits the statistical power to draw meaningful conclusions. Therefore, whether GH influences phalangeal growth in Turner patients remains unknown.

## Conclusion

We established normative reference ranges for phalangeal length ratios in females, providing a framework for evaluating phalangeal abnormalities in TS. Our data revealed differences in 4:3 MC and 5:3 MP ratios between TS and reference groups per age (or puberty) group. However, significant overlap of phalangeal bone ratios limits their clinical diagnostic utility. Larger, longitudinal studies are needed to validate these findings and assess whether combining phalangeal ratios with other skeletal markers enhances diagnostic accuracy for TS and related skeletal dysplasias.

## Data Availability

The original contributions presented in the study are included in the article/**Supplementary Material**. Further inquiries can be directed to the corresponding author.
