# Supplementary figures and images for: Phalangeal bone growth and implications in Turner syndrome
Source: Front Endocrinol (Lausanne). 2026 Jan 12;16:1735962. doi: 10.3389/fendo.2025.1735962 (PMC12832326; doi:10.3389/fendo.2025.1735962)

Supplementary Figure

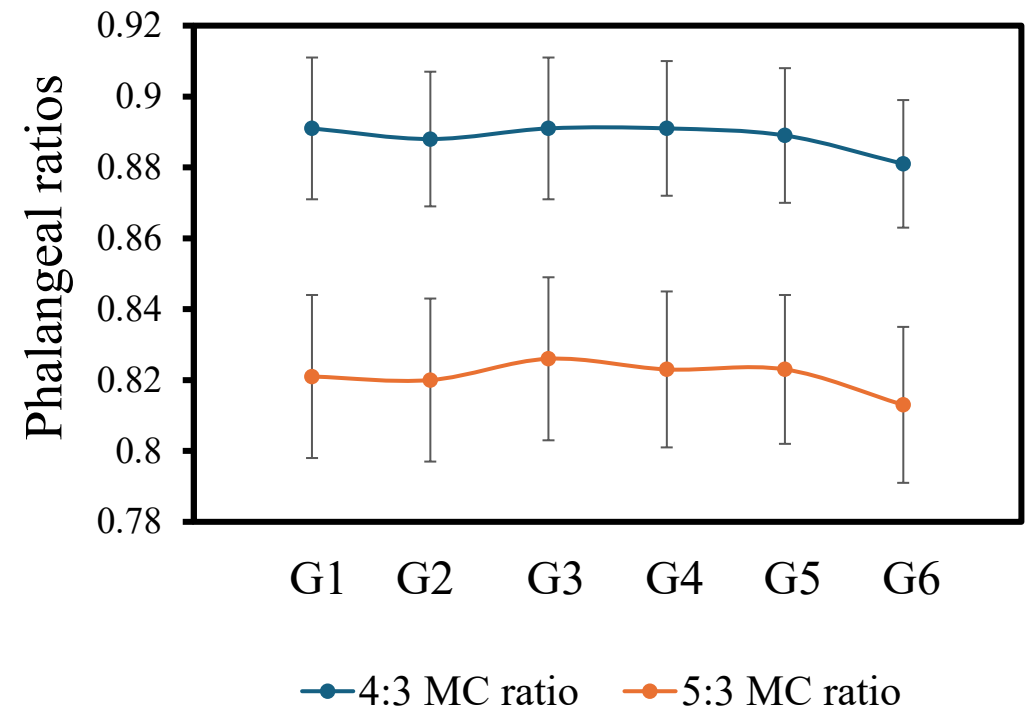

Supplement: Supplementary file 1 [file Image1.pdf]
