# Supplementary material for: Phalangeal bone growth and implications in Turner syndrome
Source: Front Endocrinol (Lausanne). 2026 Jan 12;16:1735962. doi: 10.3389/fendo.2025.1735962 (PMC12832326; doi:10.3389/fendo.2025.1735962)
Supplement: Supplementary file 2 [file Table1.pdf]

Supplemental table 1. Number of subjects in the reference and patients with Turner syndrome categorized by bone age group

Group	Bone Age (years)	Reference Number (%)	Turner syndrome Number (%)
1	0-5.9	655 (16.0)	8 (9.9)
2	6.0-7.9	775 (19.0)	14 (17.3)
3	8.0-10.9	813 (19.9)	12 (14.8)
4	11.0-12.9	820(20.1)	21 (25.9)
5	13.0-14.9	730 (17.9)	21 (25.9)
6	15.0-18.0	289 (7.1)	5 (6.2)
Total		4082 (100)	81 (100)
