# Supplementary material for: Phalangeal bone growth and implications in Turner syndrome
Source: Front Endocrinol (Lausanne). 2026 Jan 12;16:1735962. doi: 10.3389/fendo.2025.1735962 (PMC12832326; doi:10.3389/fendo.2025.1735962)
Supplement: Supplementary file 3 [file Table2.docx]

**Supplemental table 2. The reference value for three phalangeal length ratios (n = 4,082)**

| Group | 4:3 MC ratio | | | | 5:3 MC ratio | | | | 5:3 MP ratio | | | |
| --- | --- | --- | --- | --- | --- | --- | --- | --- | --- | --- | --- | --- |
|  | Mean | SD | Min | Max | Mean | SD | Min | Max | Mean | SD | Min | Max |
| 1 | 0.891 | 0.020 | 0.838 | 0.939 | 0.821 | 0.023 | 0.765 | 0.876 | 0.615 | 0.057 | 0.448 | 0.744 |
| 2 | 0.888 | 0.019 | 0.840 | 0.935 | 0.820 | 0.023 | 0.762 | 0.879 | 0.624 | 0.057 | 0.455 | 0.763 |
| 3 | 0.891 | 0.020 | 0.842 | 0.941 | 0.826 | 0.023 | 0.766 | 0.887 | 0.624 | 0.057 | 0.455 | 0.764 |
| 4 | 0.891 | 0.019 | 0.844 | 0.937 | 0.823 | 0.022 | 0.769 | 0.877 | 0.625 | 0.052 | 0.485 | 0.755 |
| 5 | 0.889 | 0.019 | 0.842 | 0.936 | 0.823 | 0.021 | 0.769 | 0.877 | 0.647 | 0.050 | 0.505 | 0.755 |
| 6 | 0.881 | 0.018 | 0.840 | 0.923 | 0.813 | 0.022 | 0.757 | 0.869 | 0.655 | 0.045 | 0.511 | 0.780 |

Abbreviations: MC, metacarpal bone; MP, middle phalangeal bone.
