# Supplementary material for: Phalangeal bone growth and implications in Turner syndrome
Source: Front Endocrinol (Lausanne). 2026 Jan 12;16:1735962. doi: 10.3389/fendo.2025.1735962 (PMC12832326; doi:10.3389/fendo.2025.1735962)
Supplement: Supplementary file 4 [file Table3.pdf]

Supplemental table 3. The prevalence of short phalangeal ratios and other skeletal abnormalities in patients with TS

	Total (n = 81)	Bone Age ≥ 8 years (n = 59)	P-value
Low 3:4 MC ratio	22 (27.1%)	15 (25.4%)	0.583
Low 3:5 MC ratio	11 (13.6%)	7 (11.9%)	0.479
Low 3:5 MP ratio	13 (16.0%)	10 (16.9%)	1.000
Brachydactyly type A3	11 (13.6%)	8 (13.6%)	1.000
Triangularization	27 (33.3%)	26 (44.1%)	< 0.001
Lucency	19 (23.5%)	18 (30.5%)	0.014
Pyramidalization	11 (13.6%)	10 (16.9%)	0.273
